# Description of Ictal HFO Mapping in Patients with Both Temporal and Extratemporal Seizure Focus

**DOI:** 10.1155/2016/5380907

**Published:** 2016-11-28

**Authors:** Juan G. Ochoa, Walter G. Rusyniak

**Affiliations:** ^1^Department of Neurology, University of South Alabama, Mobile, AL, USA; ^2^Department of Neurosurgery, University of South Alabama, Mobile, AL, USA

## Abstract

*Objective*. Review presurgical use of ictal HFO mapping to detect ictal activation areas with dual seizure focus in both the temporal and extratemporal cortex.* Methods*. Review of consecutive patients admitted to the University of South Alabama Epilepsy Monitoring Unit (SouthCEP) between January 2014 and October 2015, with suspected temporal lobe epilepsy and intracranial electrode recording. Ictal HFO localization was displayed in 3D reconstructed brain images using the patient's own coregistered magnetic resonance imaging (MRI) and computed tomography (CT) with the implanted electrodes.* Results*. Four of fifteen patients showed evidence of extratemporal involvement at the onset of the clinical seizures. Ictal HFO mapping involving both frontal and temporal lobe changed the surgical resection areas in three patients where the initial surgical plan included only the temporal lobe. Resection of the ictal HFO at the onset of the seizure and the initial propagation region was associated with seizure freedom in all patients; follow-up period ranged from 12 to 25 months.* Significance*. Extratemporal ictal involvement may not have clinical manifestations and may account for surgical failure in temporal lobe epilepsy. Ictal HFO mapping is useful to define the ictal cortical network and may help detect an extratemporal focus.

## 1. Introduction

Patients with multilobar epileptic localization that comprises the temporal lobes and surrounding areas, including the orbitofrontal cortex, the insular cortex, and the parietooccipital regions, have been described as temporal plus epilepsies (TPE) [1]. The diagnosis of TPE is rather difficult because of the similar clinical presentation with temporal lobe epilepsy (TLE). There are some features that may suggest the presence of TPE. For example, gustatory hallucinations have been associated with seizures in the insuloopercular cortex at onset; rotatory vertigo and auditory hallucinations have been associated with seizures involving the temporo-parieto-occipital junction [2]. Also, the inability to warn a seizure may suggest TPE since loss of contact has been associated with extension of the seizure out of the temporal lobe [3]. Autonomic symptoms do not clearly differentiate between TPE and TLE, but the presence of piloerection has been reported in TPE [2]. In terms of scalp EEG findings, the presence of bilateral spikes and slowing, temporoparietal or frontocentral localization was more likely associated with TPE. The diagnosis is confirmed with intracranial recording with the limitation of partial brain sampling, recording only from the implanted sites. Failure to recognize TPE is associated with poorer seizure control postoperatively. Indeed, only 8% of those who underwent standard temporal lobe surgery were seizure-free, as compared with 65% of those who benefited from resection of both temporal and extratemporal regions that were involved at seizure onset [1].

Despite advances in multimodal techniques which include MEG/EEG source imaging and fMRI, to define the epileptic zones we still do not have the technology to reliably identify the epileptic network without invasive data. In patients with intracranial recordings, the epileptic zone could be localized by the presence of persistent high frequency oscillations (HFOs). HFOs have been reported as excellent markers for the epileptogenic zone. Removal of cortical areas generating HFOs has been related to better postsurgical outcome than removing the seizure onset zone [4].

The methodology for detection of HFO and differentiation of ictal HFO versus physiological HFO is not clear. Several studies reported detection of ictal HFO with multiple band frequency analysis using different methods. One study defined ictal HFO using power spectral HFO activity during 400 ms [5]. Another study used one-second intervals starting five minutes before the onset [6]. However, the long window used in these studies does not allow the determination of the sequential cortical network activation during the ictal event that typically occurs within a much smaller time frame. Another study used the averaged 10 ms segments of similar seizures to map the seizure propagation pattern [7]. The problem is that this small window requires a high sampling rate and there is a large variability making ictal HFOs hard to differentiate from baseline. It also requires more time for analysis which makes it difficult to have the data results in time for the surgical decision making. Although fast ripples greater than 200 Hz have been correlated with the ictal zone, the HFO activity with subsequent sustained evolution has been found more robust and more spatially restricted at a peak frequency about 97 Hz [5].

The authors started using ictal HFO mapping as part of the presurgical evaluation since January 2014. We used a power spectral analysis of ictal HFO on 40-millisecond segments, synchronized with direct visualization of the standard intracranial EEG data. We compared the direct visual determination of the ictal EEG changes with the ictal HFO mapping. The surgical decision included the HFO mapping data if the epilepsy team considered resection of those areas safe and potentially beneficial for the patient.

## 2. Methods

### 2.1. Patient Population

This is a review of consecutive patients admitted to the University of South Alabama Epilepsy Monitoring Unit (SouthCEP) between January 2014 and October 2015, with suspected temporal lobe epilepsy and intracranial electrode placement to localize the epileptic zone and/or functional mapping prior resection. The typical implantation for most patients included between 30 and 64 channels including one or two 4- to 6-contact strips in the posterior orbitofrontal region, along with anterior and inferior temporal, 4- to 6-contact strips in the nondominant hemisphere, or, less commonly, a 32-contact lateral temporal grid plus anterior and inferior temporal strips in the dominant side. Additional electrodes were implanted based on noninvasive data that included scalp video EEG monitoring, scalp EEG source imaging, PET, Wada, neuropsychological testing, and 3T-MRI. Interictal and ictal EEG was recorded using the Neuvo system (Compumedics Ltd., Abbotsford, Victoria, Australia), which acquires raw data at 10 kHz and then applies a software second-order Infinite Impulse Response (IIR) Butterworth low-pass filter at 40% of the sampling frequency. For a sampling rate of 500 Hz, the resulting cutoff frequency is 200 Hz. The patient's antiepileptic medications were stopped during the intracranial monitoring in order to record sufficient seizures.

The ictal onsets were defined by initial visual inspection characterized by a clear change from the ongoing background rhythms, not explained by artifact or physiologic changes [8]. Ictal patterns were reviewed using a common average montage and standard filter setting (HFF 200 Hz, LFF 1 Hz, and notch filter 60 Hz). Simultaneously, a 40 ms segment of the same data, including the latency of the time cursor, was visualized in a synchronized view and reviewed using a band filter between 80 and 200 Hz, with notch filter on. For each time window segment, amplitude spectral power analysis (*μ*V^2^) divided by the modal frequency (Hz) was performed, with a temporal resolution of 8 ms and frequency resolution of 31 Hz. The peak of the HFO power band between 80 and 120 Hz of each segment was topographically correlated with the electrode position.

The implanted electrodes positions were obtained by manual digitization of the leads using a head CT scan after implantation and later overlaid onto an image of three-dimensional (3D) reconstructed brain images using the patient's own coregistered magnetic resonance imaging (MRI). A segment of the EEG data containing a typical seizure was then coregistered with the image data. The EEG was displayed in a common reference average montage with a constant baseline correction and filter settings (CURRY 7; Compumedics Ltd., Abbotsford, Victoria, Australia).

## 3. Results

Fifteen consecutive intracranial subdural implanted patients, with initial diagnosis of temporal lobe epilepsy, were analyzed for presence of early ictal extratemporal involvement in addition to the temporal lobes using the HFO mapping model described above. Four of these patients showed evidence of extratemporal involvement at the onset of the clinical seizures. The clinical data and HFO findings of the four patients with evidence extratemporal involvement at the onset were summarized in Table 1.

**Figure 1:**
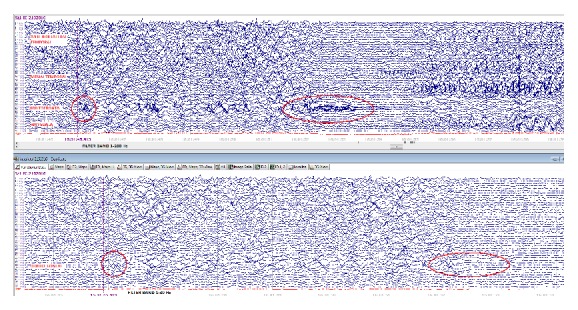
This synchronized ictal EEG on patient three at the onset of a typical seizure. The top EEG depicts interictal HFO evolving to ictal pattern. The bottom EEG only shows focal slowing at the onset but the ictal HFO is obscured by the filter.

**Figure 2:**
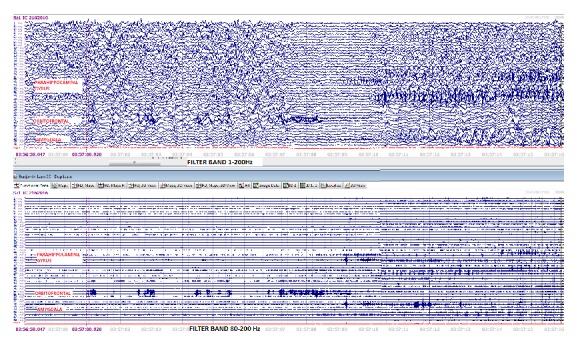
Comparison of HFO activity using a wide band (1–200 Hz) versus a ripple band (80–200 Hz).

Patient three had previous selective amygdalectomy sparing the hippocampus and failed to control her seizures. This patient was implanted for reoperation and was included in this analysis. All patients had a consistent seizure onset and initial propagation pattern of cortical ictal HFO activation in all the recorded seizures. Electrodes with inconsistent HFO activation were not considered ictal HFOs. Patient one had strong suggestion of frontal lobe involvement in addition to temporal lobe seizures by noninvasive diagnostic data. HFO mapping confirmed the extratemporal involvement at the onset of the seizures. There was no imaging or scalp EEG evidence of extratemporal involvement in the remainder of three patients. Two patients had a clear onset of ictal HFO activity in the frontal lobe before the temporal lobe (Figures 1 and 2) and patient two had temporal lobe ictal HFO activation at the onset, but the clinical seizure started with frontal HFO activity. Ictal HFO mapping helped to define the surgical resection areas in three patients where the visual inspection with standard filter band (1–70 Hz) showed unclear focal onset of the ictal activity.

**Figure 3:**
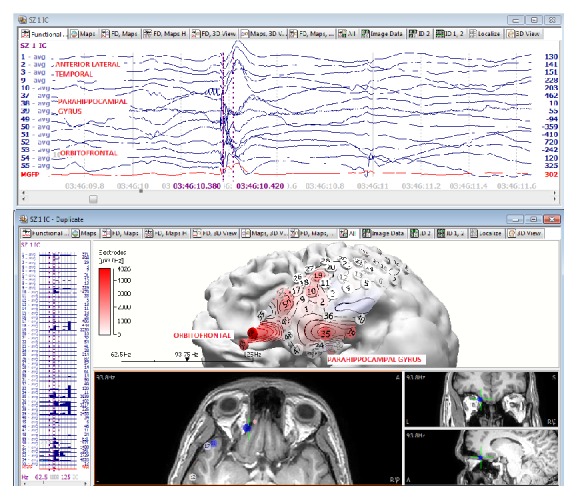
HFO mapping of initial ictal activity at the orbitofrontal and mesial temporal ictal activity of a typical seizure. The EEG data on top depicts the time window where the HFO power was calculated. The bottom image on the left side displays the graphic bar power on each EEG channel and the right side represents the localization onto a 3D model based on the patient's own MRI.

Dynamic representation of the ictal HFO activity demonstrated a back and forth sequential activation between the temporal lobe and the orbitofrontal region. This pattern of dynamic activity between the frontal and temporal lobe was not seen in the remaining eleven patients with isolated temporal lobe epilepsy (Figure 3).

Resection of the ictal HFO at the onset of the seizure and the initial propagation region was associated with seizure freedom in all patients; follow-up period ranged from 12 to 25 months.

## 4. Discussion

The epileptic network is very complex and frequently involves more than one lobe. Cortical HFO represents a relatively small area of local network activity before it is spread to a larger cortical area. Physiologic oscillations with similar frequency properties exist throughout the cortex, not related to the epileptic network, which are under the control of the thalamic pacemaker activity. Therefore only HFO activity with sustained evolution is included in this analysis. The sequential display in a short time window allows a topographical assessment of the cortical areas involved during the ictal HFO activity. This data helps us understand the epileptic network better. Consistent with previous published data, ictal HFOs can be found in distant areas from the main ictal zone [9].

In patients with multilobar involvement, the ictal zone concept does not explain well the ictal phenomenon. The data found in these small series support the idea of a complex epileptic network involving distant areas, particularly the orbitofrontal region and the temporal lobe, at times with minimal or no clinical manifestations of the multilobar involvement. Recording and analyzing intracranial EEG data with a standard filter band (1–70 Hz) and low sampling rate may prevent correct identification of the ictal HFO activity. ACNS recommends a sampling rate at least three times the high frequency filter to record a reliable activity [10]. Dynamic mapping of HFO has provided great insight about the cortical activation sequence during a seizure and identified potential critical areas that may need to be disabled either surgically or functionally to prevent new ictal development. One important limitation of this study is the lack of depth recording of the hippocampus and the subcortical pathways, giving only a partial view of the epileptic network. This series is too small to draw conclusions about the cortical areas that must be resected to permanently disable the epileptic network. Other shortcomings of this model include the limited electrode sampling, restricted to the implanted areas and the small number of subjects. Further studies with a larger population are needed to confirm these findings.

## 5. Conclusions

Intracranial ictal HFO mapping can be useful to detect ictal cortical activation areas. With adequate sampling rate and a wide filter band (1–200 Hz), focal ictal HFO activity at the onset of the seizure could be detected. This small series supports that previous publications about disabling or resecting ictal onset HFO activity are important to stop seizures. Finally, posterior orbitofrontal seizure onset may manifest as temporal epilepsy without other clinical manifestations and may be responsible for surgical failure in some cases.

## Figures and Tables

**Table 1:** Clinical features, imaging correlation, and ictal HFO data.

#	Age/gender	Interictal EEG	Ictal EEG onset	3T-MRI	Aura	Semiology	Ictal HFO mapping
1	24 y/F	R-frontal and temporal spikes	R-temporal rhythmic theta	Normal	No	Confusion, late head turn to left and left arm posturing	Right anterior frontal and right mesial temporal
2	31 y/F	Left and right temporal spikes	R-orbitofrontal followed by bitemporal	Normal	No	Generalized tonic seizure without warning	Right orbitofrontal propagating to bilateral temporal lobes
3	39 y/F	Left temporal spikes	Left anterior temporal	Larger Left hippocampus	No	Staring	Left posterior orbitofrontal region with rapid propagation to mesial temporal and subsequent lateral temporal cortex
4	27 y/M	Left temporal spikes	Left anterior temporal	Normal	Metallic smell and vision distortion	Staring followed by GTC	Left mesial temporal with rapid propagation to the posterior orbitofrontal region and subsequent lateral temporal cortex
